# Postoperative neutrophil-to-lymphocyte ratio predicts malignant cerebral edema following endovascular treatment for acute ischemic stroke

**DOI:** 10.3389/fneur.2024.1394550

**Published:** 2024-06-27

**Authors:** Sujie Zheng, Xinzhao Jiang, Shunyuan Guo, Zongjie Shi

**Affiliations:** ^1^Department of Clinical Laboratory, Laboratory Medicine Center, Zhejiang Provincial People’s Hospital (Affiliated People’s Hospital, Hangzhou Medical College), Hangzhou, Zhejiang, China; ^2^Department of Neurology, Center for Rehabilitation Medicine, Zhejiang Provincial People’s Hospital (Affiliated People’s Hospital, Hangzhou Medical College), Hangzhou, Zhejiang, China

**Keywords:** acute ischemic stroke, malignant cerebral edema, endovascular treatment, large vessel occlusion, neutrophil-to-lymphocyte ratio

## Abstract

**Background and purpose:**

Malignant cerebral edema (MCE) is one of serious complications with high mortality following endovascular treatment (EVT) for acute ischemic stroke (AIS) with large vessel occlusion. We aimed to investigate the relationship between postoperative neutrophil-to-lymphocyte ratio (NLR) and MCE after EVT.

**Methods:**

The clinical and imaging data of 175 patients with AIS of anterior circulation after EVT were studied. Admission and postoperative NLR were determined. The presence of MCE was evaluated on the computed tomography performed 24 h following EVT. The clinical outcomes were measured using the modified Rankin Scale (mRS) at 90-day after onset. Univariate and multivariate regression analyses were used to analyze the relationship between postoperative NLR and MCE. Optimal cutoff values of postoperative NLR to predict MCE were defined using receiver operating characteristic analysis.

**Results:**

MCE was observed in 24% of the patients who underwent EVT and was associated with a lower rate of favorable clinical outcomes at 90-day. Multivariate logistic regression analysis demonstrated that baseline Alberta Stroke Program Early CT Score (ASPECT) score (OR = 0.614, 95% CI 0.502–0.750, *p* = 0.001), serum glucose (OR = 1.181, 95% CI 1.015–1.374, *p* = 0.031), and postoperative NLR (OR = 1.043, 95% CI 1.002–1.086, *p* = 0.041) were independently associated with MCE following EVT for AIS with large vessel occlusion. Postoperative NLR had an area under the receiver operating characteristic curve of 0.743 for prediction MCE, and the optimal cutoff value was 6.15, with a sensitivity and specificity of 86.8% and 55%.

**Conclusion:**

Elevated postoperative NLR is independently associated with malignant brain edema following EVT for AIS with large vessel occlusion, and may serve as an early predictive indicator for MCE after EVT.

## Introduction

Malignant cerebral edema (MCE) is one of common complications with high mortality following endovascular treatment (EVT) for acute ischemic stroke (AIS) with large vessel occlusion. The mechanism and development of cerebral edema are complex, and identifying factors that influence its formation and exploring early predictive markers are of significant clinical importance for preventing the development of MCE and improving clinical outcomes.

Previous research on predicting cerebral edema after stroke has largely focused on clinical characteristics and imaging indicators, such decreased level of consciousness, nausea or vomiting, and heavy smoking, age, baseline National Institutes of Health Stroke Scale (NIHSS) score, baseline ASPECT score, collateral circulation score (1, 2). However, these indicators do not fully reflect the mechanisms underlying the development of cerebral edema. In recent years, the neutrophil-to-lymphocyte ratio (NLR), a biomarker reflecting the pathophysiological mechanisms of stroke, has been shown to be a valuable tool for predicting stroke prognosis. Brooks et al. (3) reported a significant correlation between NLR and clinical outcomes following EVT, elevated admission NLR being associated with poor outcomes and mortality. Goyal et al. (4) demonstrated a significant correlation between lower admission NLR and favorable outcomes and functional independence at 3 months for AIS patients. However, it remains unclear whether the NLR, as an inflammatory biomarker, is involved in the development of cerebral edema and whether it can serve as an early predictive marker for MCE following EVT for AIS with large vessel occlusion.

Therefore, this study aims to explore the relationship between NLR and malignant cerebral edema following endovascular treatment for AIS with large vessel occlusion.

## Materials and methods

### Study population

Patients who underwent EVT between October 2018 and November 2021 were included. Inclusion criteria were: (1) age 18 years or older; (2) confirmed occlusion of the middle cerebral artery (M1 segment) and termination of the internal carotid artery; (3) baseline NIHSS score ≥ 6, pre-stroke mRS score ≤ 2; (4) time from onset to puncture (OTP) ≤ 24 h. Exclusion criteria were: (1) documented recent infectious diseases; (2) severe cardiopulmonary dysfunction; (3) lack of complete clinical and imaging data.

This study was authorized by the Ethics Committee of Zhejiang Provincial People’s Hospital (2017KY021).

### Clinical data collection

Clinical information of the study subjects was collected, including demographic data (age and gender), medical history (hypertension, diabetes, atrial fibrillation), smoking history, stroke etiology was classified according to the Trial of ORG 10172 in Acute Stroke Treatment (TOAST), National Institutes of Health Stroke Scale (NIHSS) scores, occlusion sites, intravenous thrombolytic therapy, OTP time, number of stent retrieval passes, and recanalization status.

### Inflammatory markers and imaging evaluation

Blood samples were collected at admission and within 24 h after EVT to measure white blood cell, neutrophil, and lymphocyte counts using flow cytometry. NLR was calculated by dividing the absolute count of neutrophils by that of lymphocytes.

Computed tomography (CT) was performed at admission and 24 h after EVT. ASPECT Score was calculated at admission. Large-vessel occlusion was evaluated from the admission CT angiography. CT scans within 24 h after EVT were used to assess MCE, MCE was present if (1) >50% of the MCA area had parenchymal hypodensity with signs of local brain swelling, such as disappearance of the sulci and gyri and compression of the lateral ventricle; and (2) midline shift of ≥5 mm was present at the septum pellucidum or pineal gland with obliteration of the basal cisterns (4). Recanalization status was determined by DSA immediately after MT using the modified Thrombolysis in Cerebral Infarction (mTICI) grade. Successful recanalization was defined as an mTICI score of 2b to 3.

### Clinical outcomes

Clinical outcomes were measured using the modified Rankin Scale (mRS) at 90 days after onset. A favorable outcome was defined as an mRS of 0–2, whereas an mRS ≥ 3 was considered a poor outcome.

### Statistical analysis

The data were analyzed using the SPSS software (version 23.0; IBM, Armonk, New York). Continuous variables were reported as mean ± SD or median [interquartile range (IQR)]. Categorical variables were presented as frequencies and percentages. Continuous variables were analyzed with the student’s t test or the Mann–Whitney U-test depending on the normality of distribution. Categorical variables were analyzed with the Chi-squared test or Fisher’s exact test. Univariate and multivariate logistic regression analyses were used to evaluate the relationship between NLR and MCE. Possible confounders with a *P* < 0.05 in univariable analysis were included in the multivariable model. We used the area under the receiver operating characteristic curve to determine the best cutoff values for NLR to predict MCE. A *p*-value < 0.05 was considered statistically significant.

## Results

A total of 175 patients who underwent EVT for AIS with large vessel occlusion were included in the analysis. The median age was 70 (IQR, 58–79) years, and 106 (60.6%) patients were male. The baseline NIHSS score was 17 (IQR, 13–20), baseline ASPECTS score was 9 (IQR, 7–10), baseline NLR was 5.50 (IQR, 3.05–9.5), postoperative NLR was 6.73 (IQR, 4.33–11.14). Successful recanalization was achieved in 165 (94.3%) patients, and 47.4% participants had favorable 90-day clinical outcomes (mRS ≤ 2; Table 1).

**Table 1:** Comparison between MCE and non-MCE groups after EVT in patients with LVO.

	All patients	MCE group	Non-MCE group	*P*
	(*N* = 175)	(*N* = 42)	(*N* = 133)	
Age, years, median (IQR)	70(58–79)	73(63–80)	69(57–79)	0.204
Male, *n* (%)	106(60.6)	22(52.4)	84(63.2)	0.213
Medical history, *n* (%)				
Hypertension	120(68.2)	32(76.2)	88(66.2)	0.222
Diabetes mellitus	36(20.6)	11(26.2)	25(18.8)	0.301
Atrial fibrillation	84(48)	26(61.9)	58(43.6)	0.039
Smoking	42(24)	5(11.9)	37(27.8)	0.058
Baseline NIHSS score, median (IQR)	17(13–20)	20(17–23)	15(12–19)	0.001
Baseline ASPECT score, median (IQR)	9(7–10)	7(4–9)	10(8–10)	0.001
TOAST classification, *n* (%)				0.303
LAA	63(36)	11(26.2)	52(39.1)	
Cardioembolic	81(46.3)	23(54.8)	58(43.6)	
Undetermined or others	31(17.7)	8(28.4)	23(17.3)	
Occluded vessel site, *n* (%)				0.073
ICA	67(38.3)	21(50)	46(34.6)	
MCA-M1	108(61.7)	21(50)	87(65.4)	
OTP, median, min (IQR)	410(260–643)	377(269–573)	430(254–657)	0.731
Intravenous thrombolysis, *n* (%)	67(38.3)	11(26.2)	56(42.1)	0.064
Number of stent retrieval passes, median (IQR)	2(1–2)	2(1–3)	2(1–2)	0.166
Serum glucose, mmol/L median (IQR)	6.86(6.09–8.50)	7.51(6.58–9.53)	6.75(5.93–8.3)	0.028
Baseline NLR, median (IQR)	5.50(3.05–9.5)	9.25(4.77–12.71)	5.14(2.87–8.18)	0.001
Postoperative NLR, median (IQR)	6.73(4.33–11.14)	10.05(6.89–16.2)	5.70(4.10–8.75)	0.001
Revascularization (mTICI ≥ 2b), *n* (%)	165(94.3)	40(95.2)	125(94)	0.760
Favorable outcome, *n* (%)	83(47.4)	6(14.3)	77(57.9)	0.001

MCE, malignant cerebral edema; EVT, endovascular treatment; NIHSS, National Institutes of Health Stroke Scale; ASPECT, Alberta Stroke Program Early CT; TOAST, Trial of Org 10,172 in Acute Stroke Treatment; LAA, large-artery atherosclerosis; ICA, internal carotid artery; MCA-M1, middle cerebral artery M1 segment; OTP, time from onset to puncture; mTICI, modified Thrombolysis in Cerebral Infarction.

### Factors associated with MCE

MCE was observed in 42 (24%) patients within 24 h after the procedure. The proportion of atrial fibrillation was significantly higher in the MCE group compared to the non-MCE group (61.9% vs. 43.6%, *p* = 0.039). In the MCE group, baseline NIHSS score (20 [17–23] vs. 15 [12–19], *p* = 0.001), serum glucose (7.51 [6.58–9.53] vs. 6.75 [5.93–8.34], *p* = 0.028), baseline NLR (9.25 [4.77–12.71] vs. 5.14 [2.87–8.18], *p* = 0.001), and postoperative NLR (10.05 [6.89–16.2] vs. 5.70 [4.10–8.75], *p* = 0.001) were significantly higher than those in the non-MCE group. Conversely, baseline ASPECT score (7 [4–9] vs. 10 [8–10], *p* = 0.001) and the rate of favorable outcomes (6 [14.3] vs. 77 [57.9], *p* = 0.001) were significantly lower in the MCE group compared to the non-MCE group. No statistically significant differences were observed between the two groups in terms of age, gender, hypertension, diabetes, smoking history, TOAST classification, site of vascular occlusion, OTP time, intravenous thrombolysis, number of stent retrieval passes, and successful recanalization rate (*p* > 0.05).

After adjusting for atrial fibrillation, baseline NIHSS score, baseline ASPECT score, serum glucose, and baseline NLR, multivariate logistic regression analysis demonstrated that baseline ASPECT score (OR = 0.614, 95% CI 0.502–0.750, *p* = 0.001), serum glucose (OR = 1.181, 95% CI 1.015–1.374, *p* = 0.031), and postoperative NLR (OR = 1.043, 95% CI 1.002–1.086, *p* = 0.041) were independently associated with MCE following EVT for AIS with large vessel occlusion (Table 2).

**Table 2:** Multivariate analysis of MCE after EVT for AIS with LVO.

	*P*	OR	95%CI
Baseline NIHSS score	0.106	1.059	0.988–1.135
Baseline ASPECT score	0.001	0.614	0.502–0.750
Atrial fibrillation	0.092	2.300	0.873–6.061
Serum glucose	0.031	1.181	1.015–1.374
Postoperative NLR	0.041	1.043	1.002–1.086

MCE, malignant cerebral edema; EVT, endovascular treatment; NIHSS, National Institutes of Health Stroke Scale; ASPECT, Alberta Stroke Program Early CT.

**Figure 1:**
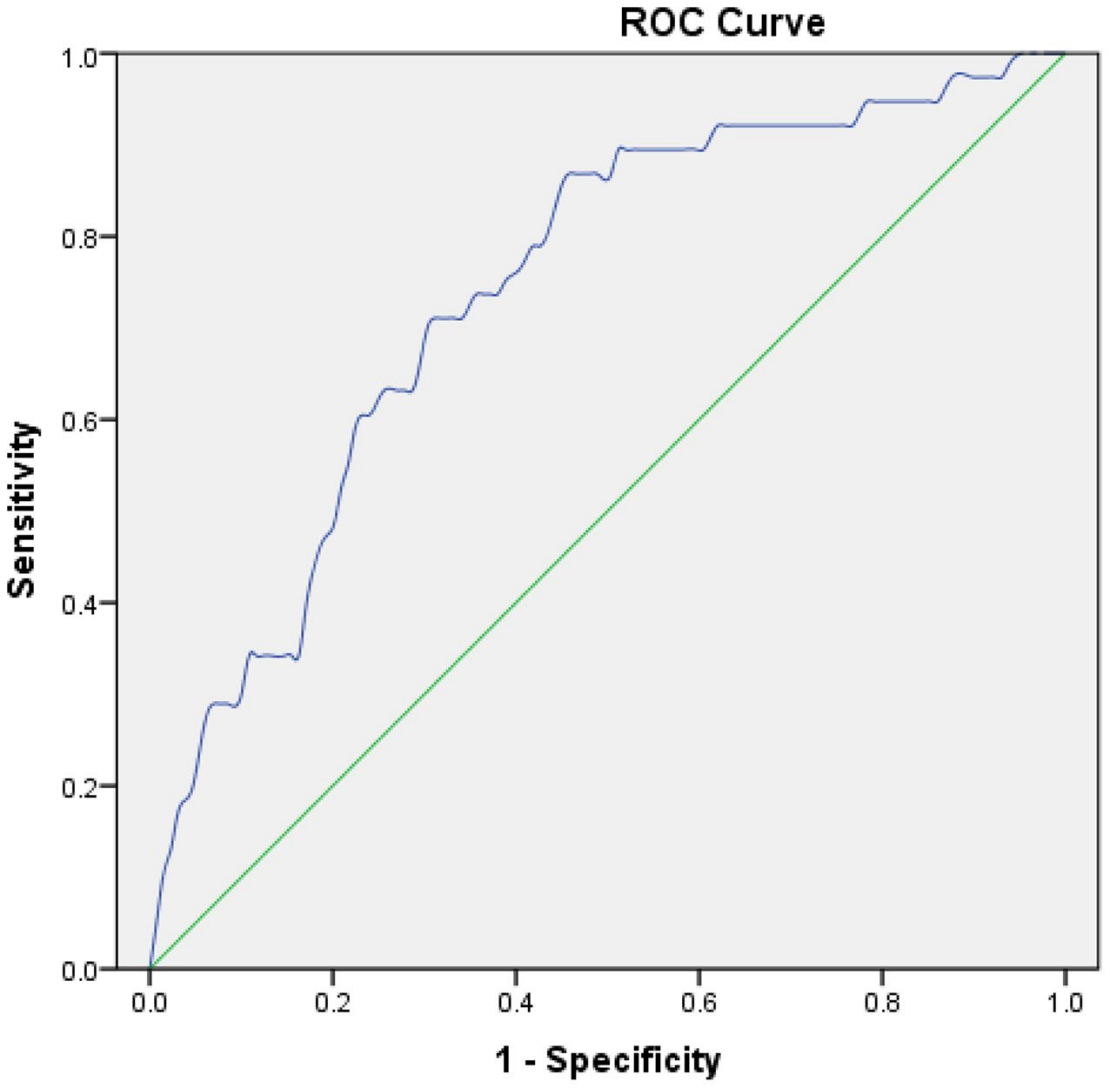
Receiver operating characteristic (ROC) curves in predicting MCE. Postoperative NLR: The area under the curve (AUC) = 0.743, cut-off = 6.15.

For postoperative NLR and MCE, postoperative NLR had an area under the receiver operating characteristic curve of 0.743, and the optimal cutoff value for postoperative NLR was 6.15 for the prediction of MCE, with a sensitivity and specificity of 86.8% and 55% when applied to an independent validation data set (Figure 1).

### Relationship between MCE and clinical outcomes

Univariate analysis revealed that the proportion of MCE in favorable outcomes group was significantly lower than poor outcomes group (7.2% vs. 39.1%, *p* = 0.001). After adjusting for age, gender, baseline NIHSS score, baseline ASPECT score, and serum glucose, logistic regression analysis demonstrated that MCE (OR = 0.169, 95% CI 0.059–0.487, *p* = 0.001) was negatively correlated with favorable outcomes at 90 days following EVT for AIS with large vessel occlusion (Tables 3, 4).

**Table 3:** Comparison between favorable outcome and poor outcome groups after EVT.

	All patients	Favorable outcome group	Poor outcome group	*P*
	(*N* = 175)	(*N* = 83)	(*N* = 92)	
Age, years, median (IQR)	70(58–79)	64(55–76)	73(64–81)	0.001
Male, *n* (%)	106(60.6)	58(69.9)	48(52.2)	0.017
Medical history, *n* (%)				
Hypertension	120(68.2)	53(63.9)	67(72.8)	0.202
Diabetes mellitus	36(20.6)	17(20.5)	19(20.7)	0.978
Atrial fibrillation	84(48)	35(42.2)	49(53.3)	0.142
Smoking	42(24)	25(30.1)	17(18.5)	0.072
Baseline NIHSS score, median (IQR)	17(13–20)	16(11–18)	18(14–23)	0.001
Baseline ASPECT score, median (IQR)	9(7–10)	9(8–10)	9(6–10)	0.046
TOAST classification, *n* (%)				0.541
LAA	63(36)	27(32.5)	36(39.1)	
Cardioembolic	81(46.3)	39(47.0)	42(45.7)	
Undetermined or others	31(17.7)	17(20.5)	14(15.2)	
Occluded vessel site, *n* (%)				0.809
ICA	67(38.3)	31(37.3)	36(39.1)	
MCA-M1	108(61.7)	52(62.7)	56(60.9)	
OTP, median, min (IQR)	410(260–643)	395(249–612)	415(266–699)	0.241
Intravenous thrombolysis, *n* (%)	67(38.3)	36(43.4)	31(33.7)	0.188
Number of stent retrieval passes, median (IQR)	2(1–2)	2(1–2)	2(1–3)	0.056
Serum glucose, mmol/L median (IQR)	6.86(6.09–8.50)	6.53(5.89–8.18)	7.25(6.28–8.87)	0.025
Revascularization (mTICI ≥ 2b), *n* (%)	165(94.3)	80(96.4)	85(92.4)	0.256
MCE, *n* (%)	83(47.4)	6(7.2)	36(39.1)	0.001

EVT, endovascular treatment; NIHSS, National Institutes of Health Stroke Scale; ASPECT, Alberta Stroke Program Early CT; TOAST, Trial of Org 10,172 in Acute Stroke Treatment; LAA, large-artery atherosclerosis; ICA, internal carotid artery; MCA-M1, middle cerebral artery M1 segment; OTP, time from onset to puncture; mTICI, modified Thrombolysis in Cerebral Infarction; MCE, malignant cerebral edema.

**Table 4:** Multivariate analysis of favorable outcome after EVT for AIS with LVO.

	*P*	OR	95%CI
Age	0.006	0.964	0.939–0.990
Male	0.548	1.255	0.598–2.633
Baseline NIHSS score	0.033	0.937	0.883–0.995
Baseline ASPECT score	0.737	1.033	0.857–1.244
Serum glucose	0.059	0.886	0.781–1.005
MCE	0.001	0.169	0.059–0.487

NIHSS, National Institutes of Health Stroke Scale; MBE, malignant brain edema; MCE, malignant cerebral edema.

## Discussion

The results of this study revealed that postoperative NLR was independently associated with MCE, and MCE was independently associated with poor 90-day outcomes following EVT for AIS with large vessel occlusion. Postoperative NLR was the best predictor of MCE following EVT, with an area under the curve around 0.743 and an optimal cutoff value of 6.15. This cutoff value showed moderate predictive accuracy with a sensitivity and specificity of 86.8% and 55% when applied to an independent validation data set.

Inflammation plays a crucial role in the pathophysiology of stroke, and elevated inflammatory markers levels have been associated with poor outcomes for AIS (5, 6). At the early stages of acute stroke, neutrophils aggregate in the ischemic area and release inflammatory mediators, leading to blood–brain barrier (BBB) disruption, increased infarct volume, hemorrhagic transformation, and poor clinical outcomes (7, 8). Lymphocytes, as primary regulators in the brain, contribute to the repair of inflammation-induced brain injury (9). The NLR, a serum biomarker that assesses the balance between neutrophils and lymphocytes, has been utilized to measure systemic inflammation and has played a critical role in predicting clinical outcomes in AIS (10, 11). Early EVT is the most effective therapeutic approach for AIS with large vessel occlusion, but it can also lead to ischemia–reperfusion injury. Reperfusion damage can cause a marked inflammatory response, further increasing brain injury. After ischemic stroke, neutrophil migration to the ischemic area, immune system activation, release of inflammatory mediators such as chemokines and cytokines, reactive oxygen species (ROS), as well as the release of adhesion molecules and proteolytic enzymes, leading to BBB disruption and increased risk of brain edema and hemorrhagic transformation (5, 6). The postoperative MCB is one of the most severe complications of EVT, leading to rapid deterioration of neurological function and poor clinical outcomes. Based on these findings, we hypothesize that inflammation contributes to BBB disruption, triggering or promoting MCE, contributing to poor clinical outcomes. NLR as a representative biomarker of inflammation, has not been fully evaluated for early prediction of MCE following early EVT in previous studies. In this study found that postoperative NLR was independently associated with MCE following early EVT for AIS with large vessel occlusion.

Furthermore, the timing of measuring NLR deserves clinical attention. Bi et al. (12) indicated that an increased admission NLR was associated with poor outcomes at 3 months for AIS patients receiving EVT. Sharma et al. (13) demonstrated that lower admission NLR was associated with favorable outcomes. Wu et al. (14) demonstrated that admission and post-EVT NLR were associated with poor outcomes, but postoperative NLR showed better predictive ability for poor outcomes. Consistent with these previous findings, our study found that NLR at admission and within 24 h post-EVT were associated with early MCE following EVT, but multivariate analysis showed that only postoperative NLR values were independently associated with MCE. It is speculated that inflammatory markers measured in blood samples after reperfusion therapy may more accurately reflect stroke-related immune responses. Neutrophils infiltrate the ischemic area within 30 min to several hours post-stroke, peaking at 1–3 days post-stroke and subsequently declining steadily (15). Regulatory lymphocyte levels in the ischemic area are low on the first day post-stroke (16). BBB disruption after reperfusion leads to a rapid increase in vasogenic brain edema at 24–48 h after reperfusion. Therefore, dynamic monitoring of NLR may be more beneficial for predicting early MCE, hemorrhagic transformation, and poor outcomes following EVT.

In the acute phase of ischemic stroke, blood glucose can infiltrate the damaged BBB, increase the osmotic pressure of ischemic brain tissue, promote lactate production, and exacerbate brain injury. Previous studies have found associations between elevated blood glucose levels, history of diabetes, and poor clinical outcomes (17–19). Rinkel et al. (20) demonstrated that admission high blood glucose level was associated with poor outcomes and symptomatic intracerebral hemorrhage in acute ischemic stroke patients receiving EVT. Cannarsa et al. (21) found that stress-induced hyperglycemia may be a better predictor of clinical outcomes than absolute blood glucose elevation. Recently, the relationship between blood glucose and brain edema following EVT has garnered attention. Broocks et al. (22) found a significant relationship between high blood glucose at admission and brain edema following EVT. Consistent with previous studies, our study found that high blood glucose at admission was independently associated with MCE following EVT. Stress-induced hyperglycemia following AIS is caused by acute increases in catecholamines, cortisol, and inflammatory cytokines, leading to increased hepatic glucose output (23). Research indicates that high blood glucose can harm AIS through various pathways, including endothelial dysfunction, oxidative stress, relative insulin deficiency, increased reperfusion injury, and increased risk of hemorrhagic transformation and brain edema (24–29). Therefore, dynamic monitoring of perioperative blood glucose levels and rational control of blood glucose levels are important for preventing the MCE following EVT and improving clinical outcomes.

Previous studies have found associations between younger age, baseline NIHSS score, baseline ASPECT score, collateral circulation score, and unsuccessful recanalization and MCE following EVT for AIS with large vessel occlusion (1). Additionally, dynamic measuring of inflammatory biomarkers such as NLR can improve the comprehensiveness and accuracy of early prediction of MCE following EVT. Explore new biomarkers of inflammation, clarification of their pro-inflammatory pathways and related mechanisms, and evaluation of the role of immunomodulatory drugs in early intervention for MCE in clinical trials will be beneficial for improving the clinical outcomes of EVT for AIS with large vessel occlusion.

Limitations of this study include: (1) it is single-center retrospective design, which may introduce biases that cannot be completely avoided. (2) assessment of baseline infarct volume using ASPECTS scoring may not be precise enough, potentially impacting the final study results. Future studies will incorporate CT or MR perfusion imaging for quantitative assessment of baseline infarct volume. (3) collateral circulation score may be related to postoperative brain edema, and may enhance the accuracy of early prediction of MCE following EVT. Our study did not include collateral scores.

In conclusion, elevated postoperative NLR is independently associated with malignant brain edema following EVT for AIS with large vessel occlusion, and may serve as an early predictive indicator for MCE after EVT.

## Data availability statement

The original contributions presented in the study are included in the article/supplementary material, further inquiries can be directed to the corresponding authors.

## Ethics statement

The studies involving humans were approved by Ethics Committee of Zhejiang Provincial People’s Hospital (2017KY021). The studies were conducted in accordance with the local legislation and institutional requirements. Written informed consent for participation in this study was provided by the participants’ legal guardians/next of kin. Written informed consent was obtained from the individual(s) for the publication of any potentially identifiable images or data included in this article.

## Author contributions

SZ: Data curation, Formal analysis, Writing – original draft, Writing – review & editing. XJ: Data curation, Formal analysis, Writing – original draft, Writing – review & editing. SG: Data curation, Formal analysis, Investigation, Methodology, Supervision, Writing – review & editing. ZS: Formal analysis, Investigation, Methodology, Supervision, Writing – review & editing.
